# The effects of hyperbaric oxygen therapy on oxidative stress, inflammation, and symptoms in children with autism: an open-label pilot study

**DOI:** 10.1186/1471-2431-7-36

**Published:** 2007-11-16

**Authors:** Daniel A Rossignol, Lanier W Rossignol, S Jill James, Stepan Melnyk, Elizabeth Mumper

**Affiliations:** 1 International Child Development Resource Center, 3800 West Eau Gallie Blvd., Suite 105, Melbourne, FL, 32934, USA; 2 University of Arkansas for Medical Sciences, Department of Pediatrics, Arkansas Children's Hospital Research Institute, 1120 Marshall St., Little Rock, AR 72202, USA; 3 Advocates for Children, Ltd., 2015 Tate Springs Rd., Lower Level, Suite 2, Lynchburg, VA 24501, USA; 4 Current address: 2919 Confederate Ave. Lynchburg, VA 24501, USA

## Abstract

**Background:**

Recently, hyperbaric oxygen therapy (HBOT) has increased in popularity as a treatment for autism. Numerous studies document oxidative stress and inflammation in individuals with autism; both of these conditions have demonstrated improvement with HBOT, along with enhancement of neurological function and cognitive performance. In this study, children with autism were treated with HBOT at atmospheric pressures and oxygen concentrations in current use for this condition. Changes in markers of oxidative stress and inflammation were measured. The children were evaluated to determine clinical effects and safety.

**Methods:**

Eighteen children with autism, ages 3–16 years, underwent 40 hyperbaric sessions of 45 minutes duration each at either 1.5 atmospheres (atm) and 100% oxygen, or at 1.3 atm and 24% oxygen. Measurements of C-reactive protein (CRP) and markers of oxidative stress, including plasma oxidized glutathione (GSSG), were assessed by fasting blood draws collected before and after the 40 treatments. Changes in clinical symptoms, as rated by parents, were also assessed. The children were closely monitored for potential adverse effects.

**Results:**

At the endpoint of 40 hyperbaric sessions, neither group demonstrated statistically significant changes in mean plasma GSSG levels, indicating intracellular oxidative stress appears unaffected by either regimen. A trend towards improvement in mean CRP was present in both groups; the largest improvements were observed in children with initially higher elevations in CRP. When all 18 children were pooled, a significant improvement in CRP was found (p = 0.021). Pre- and post-parental observations indicated statistically significant improvements in both groups, including motivation, speech, and cognitive awareness (p < 0.05). No major adverse events were observed.

**Conclusion:**

In this prospective pilot study of children with autism, HBOT at a maximum pressure of 1.5 atm with up to 100% oxygen was safe and well tolerated. HBOT did not appreciably worsen oxidative stress and significantly decreased inflammation as measured by CRP levels. Parental observations support anecdotal accounts of improvement in several domains of autism. However, since this was an open-label study, definitive statements regarding the efficacy of HBOT for the treatment of individuals with autism must await results from double-blind, controlled trials.

**Trial Registration:**

clinicaltrials.gov NCT00324909

## Background

Autism is a neurodevelopmental disorder currently affecting as many as 1 out of 150 individuals in the United States [1]. Autism is characterized by impairments in social interaction, difficulty with communication, and restrictive and repetitive behaviors [2]. Autism traditionally is considered a "static" neurological disorder [3] and improvements in core autistic features are not common [4,5]. Furthermore, three rigorously performed epidemiological studies demonstrate that the prevalence of autism has increased in recent years [6-8]. These facts might explain why parents of children with autism are more likely to seek alternative and off-label medical therapies than parents of children in the general population [9]. One off-label therapy that has recently increased in popularity as a treatment for autism is hyperbaric oxygen therapy (HBOT) [10,11]. Traditionally, HBOT involves inhaling up to 100% oxygen at a pressure greater than one atmosphere (atm) in a pressurized chamber [12]. Most typical indications for HBOT involve the use of hyperbaric pressures above 2.0 atm. Higher atmospheric pressures are generally required to treat conditions such as carbon monoxide poisoning and to improve wound healing [12,13].

In some studies, the use of oxygen appears to enhance neurological function. For instance, in a double-blind, placebo-controlled, cross-over study, oxygen administration in healthy young adults, when compared to room air, was demonstrated to enhance cognitive performance, including improved performance on attention, reaction times, and word recall [14]. Additionally, in elderly patients, HBOT at 2.5 atm and 100% oxygen, when compared to a control group, improved cognitive function, including memory [15]. Because of these outcomes, some investigators have used HBOT to treat certain neurological disorders, including chronic and traumatic brain injury [16-22], as well as fetal alcohol syndrome [23], and clinical improvements in these patients have been observed. Furthermore, in a recent rat model of traumatic brain injury, treatment with HBOT at 1.5 atm and 100% oxygen, when compared to a sham-treated normobaric air group, improved spatial learning and memory [24]. Several studies, using HBOT at similar pressures, also demonstrated clinical improvements in some patients with cerebral palsy (CP) [25-28] that in some cases was dramatic [29]; however, some researchers have questioned the results of these studies and have called for further controlled trials and a focus on defining the mechanism of action of HBOT in individuals with CP [30]. It is important to note that some of these studies [16,21-24,26] used lower hyperbaric pressures (1.5 atm or less) than the pressures typically used for most clinical indications [13]. Given this background, some physicians have also applied similar lower hyperbaric pressures of 1.3 to 1.5 atm in autistic individuals, with oxygen concentrations ranging from 21% to 100% [10,31].

HBOT for children is generally regarded as safe, even at pressures of 2.0 atm for 2 hours per day [32]. However, to our knowledge, the safety of HBOT for autistic children has not been previously studied; a review of MEDLINE indicates that there are no prospective studies on the use of HBOT for autism. Yet, there are anecdotal reports of clinical improvements in autistic children with hyperbaric therapy that have been reported by some physicians. For instance, Heuser et al. treated a four year old child with autism using hyperbaric therapy at 1.3 atm and 24% oxygen and reported "striking improvement in behavior including memory and cognitive functions" after only ten sessions. This child also had marked improvement of cerebral hypoperfusion as measured by pre-hyperbaric and post-hyperbaric Single Photon Emission Computed Tomography (SPECT) scans [31]. Another case series suggested that hyperbaric therapy at 1.3 atm led to clinical improvements in six autistic children [10].

Review of the pathophysiology found in some autistic individuals in conjunction with the mechanisms of action of HBOT lead to the speculation that HBOT might produce clinical improvements in autistic individuals [11]. Several studies indicate that some autistic individuals manifest cerebral hypoperfusion [33-35], neuroinflammation [36-38], and gastrointestinal inflammation [39,40]. HBOT might ameliorate some of these problems by improving cerebral hypoperfusion [17,21,31,41], and by decreasing neuroinflammation and gastrointestinal inflammation [42-47]. However, no prospective studies have examined the role of HBOT on inflammation and cerebral hypoperfusion in autistic individuals.

Furthermore, concerns exist that HBOT might increase oxidative stress via the production of reactive oxygen species [48]. These concerns are especially relevant because some children with autism express evidence of increased oxidative stress [49] including lower serum glutathione levels [50,51], and decreased activities of antioxidant enzymes including superoxide dismutase (SOD) [52], glutathione peroxidase [52], catalase [53], and paraoxonase, an enzyme that prevents lipid oxidation and also inactivates organophosphate toxins in humans [54]. Some autistic children also demonstrate evidence of increased lipid peroxidation [53,55,56]; this includes increased malondialdehyde which is a marker of oxidative stress and lipid peroxidation [57]. A review of the literature indicates that oxidative stress can occur with HBOT but appears to be less of a concern at hyperbaric pressures under 2.0 atm [58]. In fact, with long-term and repeated administration, HBOT below 2.0 atm can actually decrease oxidative stress [59-61] by reducing lipid peroxidation [62], and by up-regulating the activity of antioxidant enzymes including SOD [60,63], glutathione peroxidase [64], catalase [65], and paraoxonase [62,66]. Furthermore, at the pressures examined in this current study (1.3 to 1.5 atm), a search of the literature failed to identify any studies indicating that oxidative stress worsened with HBOT.

Alternatively, some evidence suggests that HBOT could actually alleviate oxidative stress in children with autism. For example, halving oxygen concentrations in normal healthy volunteers results in relative hypoxia and actually increases oxidative stress [67]. There are several studies that demonstrate evidence of cerebral hypoxia, as measured by a reduction in brain Bcl-2 and an increase in brain p53, among some autistic individuals [68-71]. Elevated p53 is induced by hypoxia [72] and a decrease in Bcl-2 is associated with increased apoptosis provoked by hypoxia [73]. Therefore, in theory, improving hypoxic areas in the autistic brain might decrease oxidative stress. However, the effects of HBOT on oxidative stress in autistic individuals are unknown. To our knowledge, there have been no studies performed which examine the role of HBOT on oxidative stress in autistic children.

This present study examined hyperbaric therapy at the low and the high ends of the ranges of atmospheric pressures and oxygen concentrations currently employed in individuals with autism: 1.3 atm and 24% oxygen [31], and 1.5 atm and 100% oxygen. This study had several objectives. First, since increased oxidative stress is found in some autistic children, the effects of HBOT on oxidative stress markers before and after 40 hyperbaric treatments were measured. Second, evidence of increased inflammation is found in many autistic individuals. HBOT is also known to have anti-inflammatory effects; therefore, the impact of HBOT on an inflammatory marker (C-reactive protein) was measured. Third, since the efficacy of HBOT in autism has not been previously evaluated, this current open-label pilot study (without a placebo-control group) examined the changes in clinical symptoms, as rated by parents or caregivers, after treatment with HBOT. Finally, the safety of HBOT, used at 1.3 and 1.5 atm, was evaluated in autistic children.

## Methods

### Patients

Eighteen children, 4 girls and 14 boys, ages ranging 3 to 16 years, were assessed for participation and enrolled in the study. Six children were non-randomly assigned to 1.5 atm and 100% oxygen, and the 12 remaining children were non-randomly assigned to 1.3 atm and 24% oxygen. This unequal division of children among the sample groups occurred due to scheduling constraints and because one center (EM) only treated the 1.3 atm group (6 children) while the other center (DR) treated both the 1.3 atm (6 children) and the 1.5 atm (6 children) groups. All participants were diagnosed with autistic disorder from an independent psychologist, neurologist, psychiatrist, or developmental pediatrician and met the DSM IV criteria for autistic disorder [2]. Children with a diagnosis of Pervasive Developmental Disorder – Not Otherwise Specified (PDD-NOS) or Asperger Syndrome were excluded from this study. Children with a history of seizure disorder were also excluded. Written informed consent was obtained from the parents and, when possible, the child. The study and protocol were approved by the Liberty Institutional Review Board. Baseline Childhood Autism Rating Scale (CARS) scores were obtained to determine autism severity; degrees of autism were similar in both groups (see Table 1). During the study period, children were not allowed to begin any new therapies or stop any current therapies, including medications and supplements. The children in this study were recruited from two practices (DR and EM) in which antioxidant use and treatments to raise glutathione levels are common therapies. Because of this, many of the children were already taking supplements before the study began, such as folinic acid or methylcobalamin (see Table 1). No significant differences in supplement usage, age, or initial CARS score were found between the children in the 1.5 atm group as compared to the 1.3 atm group.

**Table 1:** Baseline participant characteristics and supplement profiles

	1.3 atm group	1.5 atm group	Comparison between groups (p-value)
**A. Child characteristics**			
Age Range	3–16	3–16	
Mean Age	6.2 ± 4.0	7.7 ± 4.5	NS
Mean initial CARS score	33.8 ± 6.3	34.4 ± 8.0	NS
			
**B. Percentage of children on supplement**			
Multivitamin	92%	100%	NS
Minerals	75%	67%	NS
Digestive Enzymes	42%	17%	NS
Probiotics	50%	17%	NS
Omega-3 fatty acids	92%	100%	NS
Methylcobalamin	58%	83%	NS
Folinic acid	42%	83%	NS
Glutathione	25%	50%	NS

NS = not statistically significant

### Hyperbaric treatment protocol at 1.3 atm and 24% oxygen

Twelve children (11 boys and 1 girl, mean age 6.2 ± 4.0 years, range 3–16 years) were assigned to separately receive hyperbaric therapy at approximately 1.3 atm and 24% oxygen in a monoplace hyperbaric chamber. Each child entered the chamber with a parent or other caregiver. Compression time to obtain a pressure of 1.3 atm was approximately 10 minutes. During this time the children equilibrated their middle ears by swallowing liquid, eating, or yawning. Oxygen at 10 liters per minute from an oxygen concentrator was mixed with room air and pumped into the chamber. This resulted in a final chamber oxygen concentration of approximately 24% as measured by an oxygen monitor. The child was monitored during the entire treatment cycle. After 45 minutes of 24% oxygen at 1.3 atm, the chamber was decompressed over approximately 10 minutes. This therapy was given 45 minutes daily for an average of 4.6 times per week over an average of a 9.0 week period, for a total of 40 treatments per child.

### Hyperbaric treatment protocol at 1.5 atm and 100% oxygen

Six children (3 boys and 3 girls, mean age 7.7 ± 4.5 years, range 3–16 years) were assigned to separately receive hyperbaric therapy at 1.5 atm and 100% oxygen in a monoplace hyperbaric chamber. Each child entered the chamber with a parent or other caregiver. Compression time to obtain a pressure of 1.5 atm was approximately 15 minutes. During this time, the children equilibrated their middle ears by swallowing liquid, eating, or yawning. Each child was fitted with a rubber-neck collar and clear plastic hood through which 100% oxygen was delivered. The rubber-neck collar was applied before getting into the chamber and the plastic hood was attached after a pressure of 1.5 atm was attained. Two hoses, one for oxygen input and one for oxygen exit, were then attached to the hood. The oxygen was then turned on and entered the hood through one hose and exited through the second hose and was vented to outside the chamber. The chamber was pressurized with room air and the oxygen concentration of the chamber remained below 23% during the course of the treatment. The child was monitored during the entire treatment cycle. After 45 minutes of 100% oxygen at 1.5 atm, the oxygen was turned off, the hood was removed, and the chamber was decompressed over approximately 10 minutes. This therapy was given 45 minutes daily for an average of 4.7 times per week over an average of an 8.8 week period, for a total of 40 treatments per child.

### Blood for C-reactive protein and oxidative stress markers

Immediately prior to the first hyperbaric treatment and within 24 hours of finishing the 40^th ^(last) hyperbaric treatment, fasting blood specimens for measuring C-reactive protein (CRP) and oxidative stress profiles were drawn. The oxidative stress profiles were obtained and analyzed by SJJ and SM in a blinded fashion according to procedures previously described [50,51]. The CRPs were sent to LabCorp for analysis. The technicians at LabCorp were blinded to the fact that any of the submitted samples were for use in this study, and the same laboratory instrumentation and techniques were used to measure the before and after CRP samples.

### Clinical outcome measures

Pre-treatment scores and post-treatment scores were calculated for each child using the Aberrant Behavior Checklist – Community (ABC-C), Social Responsiveness Scale (SRS), and the Autism Treatment Evaluation Checklist (ATEC). To determine outcomes, a parent or other caretaker filled out each scale prior to treatment, and after 10, 20, 30, and 40 hyperbaric sessions.

The ABC-C is a 58-item questionnaire that assesses communication, reciprocal social interaction, play, and stereotyped behaviors [74]. It is used to evaluate the effects of medications and other therapeutic interventions and is scored from 0 ("not at all a problem") to 3 ("problem is severe in degree"). The ABC-C is widely and successfully used in clinical trials of autistic individuals [75,76]. For this study, in addition to scores in 5 subsets (irritability, social withdrawal (also termed lethargy), stereotypy, hyperactivity, and inappropriate speech), an overall score was also calculated.

The SRS is a recently validated test of interpersonal behavior, communication, and stereotypical traits in autism [77]. It consists of five subscales: social awareness, social cognition, social communication, social motivation, and autistic mannerisms. The SRS measures the degree of social impairments in autistic children and is suitable for assessing treatment outcomes. In this study, a total raw score was obtained and raw scores were calculated for each subscale.

The ATEC is a questionnaire that was developed by the Autism Research Institute to evaluate treatment efficacy in autistic individuals. It consists of four subscales labeled: Speech/Language/Communication, Sociability, Sensory/Cognitive Awareness, and Health/Physical/Behavior. The scores are weighted according to the response and the corresponding subscale. The higher the subscale and total scores, the more impaired the subject. A split-half reliability analysis on 1,358 checklists indicated high internal consistency among the questions within each subscale [78]. ATEC is used in some studies as an outcome measure [79,80]. It is designed to allow parents and physicians to assess outcomes of certain treatments commonly used in autistic individuals. In this study, scores were calculated for the total score and the four separate subscales.

### Safety Assessments

In descending order, the most common side effects found during HBOT are barotrauma (2% incidence), sinus squeeze, serous otitis, claustrophobia, reversible myopia, and new onset seizure (which occurs in 1–3 per 10,000 treatments) [12]. Before beginning the study, each child underwent a physical examination by either DR or EM; this included close examination of the ears and tympanic membranes. During each treatment, a parent or caregiver entered the chamber with each child. Throughout the treatment, children were monitored closely by the chamber operator for any signs of ear pain, and parents were instructed on how to recognize ear pain in their child. One child in the 1.5 atm group could not tolerate the pressure given during the first HBOT session, and the treatment had to be stopped after just several minutes (the pressure obtained in this session was approximately 1.1 atm). Examination of the child's ears did not demonstrate any barotrauma. However, the child's typanostomy tubes had recently fallen out; these were replaced before continuing the trial, and the child was able to finish 40 treatments without further incident. No other adverse events were found during this study, including barotrauma or seizures. All children finished 40 hyperbaric treatments.

### Data analysis

All data are presented as means ± SDs. The data were prospectively collected and analyzed using SigmaStat software. Statistical differences in changes in each scale (ABC-C, SRS, and ATEC) and changes in CRP and oxidative stress markers between baseline versus end of 40 hyperbaric treatments were ascertained using the Student's *t *test with significance set at 0.05.

## Results

### Oxidative stress profiles

**Figure 1:**
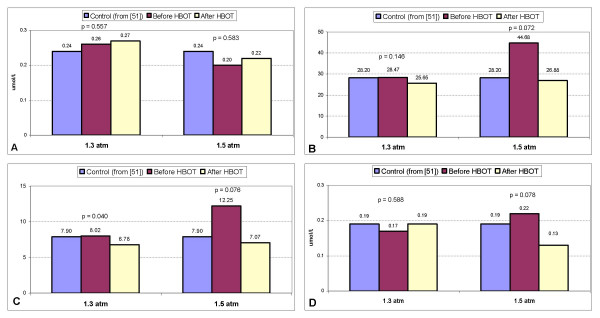
Changes in mean blood values before and after hyperbaric therapy at both 1.3 atm and 1.5 atm. The first column in a-d is the mean value for control children as described by James et al. [51] and is included as a standard reference (labeled as "control"). P-values and blood levels are listed above the bar graphs. **a**: Changes in mean oxidized glutathione levels. **b**: Changes in mean tGSH/GSSG.**c**: Changes in mean fGSH/GSSG. **d**: Changes in mean adenosine levels

Figure 1(a–d) lists the oxidative stress profile findings; the first column in each graph is the mean value for control children as described by James et al. [51] and is included as a standard reference (labeled as "control"). Mean plasma oxidized glutathione (GSSG) did not significantly change in either the 1.3 atm group (p = 0.557) or the 1.5 atm group (p = 0.583). Total plasma glutathione (tGSH) to GSSG ratio (tGSH/GSSG) (p = 0.146 at 1.3 atm; p = 0.072 at 1.5 atm) and free glutathione (fGSH) to GSSG ratio (fGSH/GSSG) (p = 0.040 at 1.3 atm; p = 0.076 at 1.5 atm) both decreased after HBOT at 1.3 atm and 1.5 atm. Mean adenosine slightly increased at 1.3 atm (p = 0.588), and decreased at 1.5 atm (p = 0.078).

### CRP profiles

**Figure 2:**
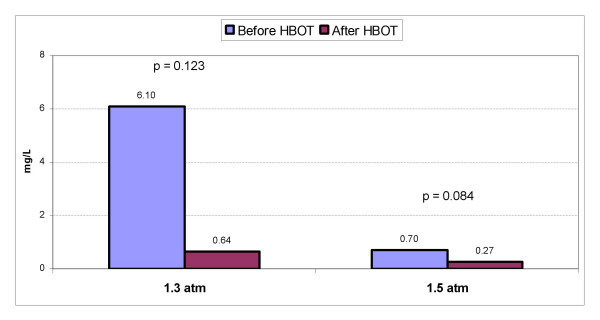
Changes in mean CRP before and after hyperbaric therapy at both 1.3 atm and 1.5 atm. P-values and blood levels are listed above the bar graphs.

Figure 2 shows the changes in mean CRP in both groups. In the 1.3 atm group, mean CRP level declined by 89.5% from 6.1 ± 10.3 mg/L to 0.64 ± 0.87 mg/L (p = 0.123). Of note, 3 children had a mean starting CRP value of 21.8 ± 9.2 mg/L ("high CRP group"), which declined to 0.2 mg/L in each child (p = 0.052) after hyperbaric therapy. Analysis of the remaining 9 children ("low CRP group") demonstrated no significant change in mean CRP values (0.88 mg/L to 0.79 mg/L, p = 0.854). In the 1.5 atm group, mean CRP declined by 61.4% from 0.7 ± 0.5 mg/L to 0.27 ± 0.19 mg/L (p = 0.084). Examination of CRP in all 18 children in the study demonstrated that CRP declined by 88.4% from a mean starting value of 4.3 ± 8.7 mg/L to 0.5 ± 0.7 mg/L (p = 0.021).

### Clinical Outcomes

#### 1.3 atm group analysis

**Figure 3:**
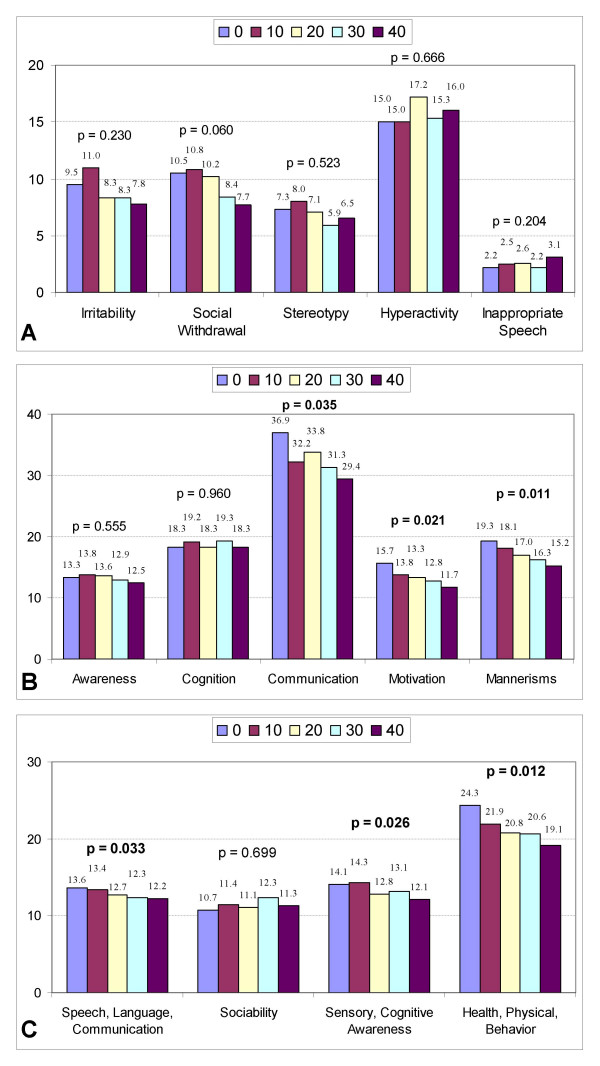
Changes in clinical scales at 1.3 atm and 24% oxygen. Declining scores on each scale indicate clinical improvements. Scores are listed above the bar graphs at baseline (0) and after every 10 treatments (10, 20,30, and 40). P-values are listed above the bar graphs. **a**:Changes in ABC-C subscales at 1.3 atm and 24% oxygen. **b**:Changes in SRS subscales at 1.3 atm and 24% oxygen. **c**: Changes in ATEC subscales at 1.3 atm and 24% oxygen.

Table 2 shows improvements in SRS (p = 0.046) and ATEC (p = 0.007) for the 12 children in the 1.3 atm group. Evaluation of the ABC-C, SRS, and ATEC subscales (Figure 3a–c) demonstrates significant improvements in SRS communication (p = 0.035); SRS motivation (p = 0.021); SRS mannerisms (p = 0.011); ATEC speech/language/communication (p = 0.033); ATEC sensory/cognitive awareness (p = 0.026); and ATEC health/physical/behavior (p = 0.012).

**Table 2:** Aggregate mean scores for 12 children at 1.3 atm, 24% oxygen

**1.3 atm**	Mean Score Before HBOT	Mean Score After HBOT	Percentage Improvement	p-value
ABC-C	44.4 ± 22.0	40.2 ± 21.5	9.5	0.458
SRS	104.3 ± 29.8	87.1 ± 22.9	16.5	**0.046**
ATEC	61.4 ± 20.8	54.6 ± 17.2	11.1	**0.007**

#### 1.5 atm group analysis

**Figure 4:**
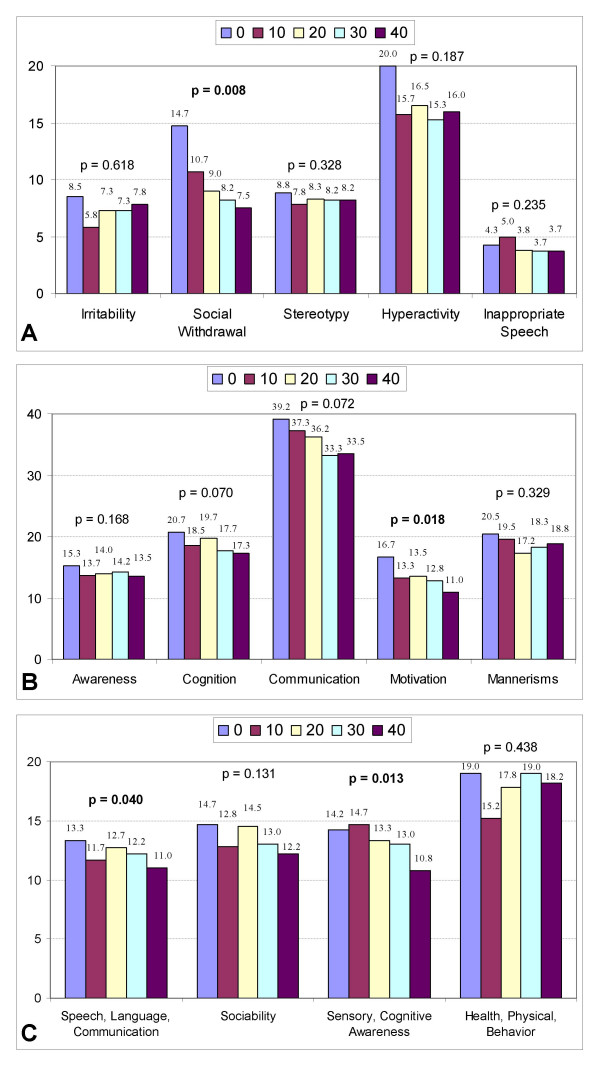
Changes in clinical scales at 1.5 atm and 100% oxygen. Declining scores on each scale indicate clinical improvements. Scoresare listed above the bar graphs at baseline (0) and after every 10 treatments (10, 20, 30, and 40). P-values are listed above the bar graphs. **a**: Changes in ABC-C subscales at 1.5 atm and 100% oxygen. **b**: Changes in SRS subscales at 1.5 atm and 100% oxygen. **c**: Changes in ATEC subscales at 1.5 atm and 100% oxygen.

Table 3 shows improvements in SRS (p = 0.035) and ATEC (p = 0.020) for the 6 children in the 1.5 atm group. Examination of the subscales (Figure 4a–c) demonstrates significant improvements in ABC-C social withdrawal (p = 0.008); SRS motivation (p = 0.018); ATEC speech/language/communication (p = 0.040); and ATEC sensory/cognitive awareness (p = 0.013).

**Table 3:** Aggregate mean scores for 6 children at 1.5 atm, 100% oxygen

**1.5 atm**	Mean Score Before HBOT	Mean Score After HBOT	Percentage Improvement	p-value
ABC-C	56.3 ± 27.3	43.2 ± 25.9	23.3	0.094
SRS	112.3 ± 30.9	95.0 ± 38.9	15.4	**0.035**
ATEC	61.2 ± 28.0	52.2 ± 28.0	14.7	**0.020**

## Discussion

To our knowledge, this study represents the first prospective study on the use of HBOT for children with autism. In this study, lower hyperbaric pressures were used than those traditionally employed (typically pressures of 2.0 atm and above [13]) for the treatment of most clinical indications. However, significant increases in oxygen delivery were obtained during this study. The oxygen concentration in room air at sea level (1 atm) is about 160 mmHg. The two study sites were located at approximately 500 and 900 feet above sea level (0.97–0.98 atm). Therefore, the oxygen delivery in the 1.3 atm group was approximately 232 mmHg which is roughly 45% more than room air conditions. In the 1.5 atm group, the oxygen delivery was 1142 mmHg, or over 7 times more than room air conditions. The amount of oxygen delivered in the 1.3 atm group is similar to the amount used in a previous study on HBOT in children with CP that utilized 1.3 atm and room air pressure ("hyperbaric air") [26]. In that study, the authors commented that the amount of oxygen delivered at 1.3 atm was achievable with the use of "28% oxygen with a mask, without pressure"; however, this opinion did not account for the potential clinical effects of the increased atmospheric pressure delivered, because even low amounts of increased pressure may lead to significant clinical changes [44,81]. Furthermore, the authors argued that hyperbaric air at 1.3 atm was unlikely to provide clinical benefit(s) because the mechanism of action of HBOT in CP is thought to be due to the "penumbra phenomenon" and that a clinical effect due to "a pure pressure effect" did "not correspond to the rationale behind the hyperbaric oxygen treatment" [26]. Since the mechanism of action of HBOT in autism may be different than in CP [11], including decreasing inflammation (as reviewed in the background section of this paper), it is entirely possible that clinical benefits may arise from purely increasing the atmosphere pressure delivered, because increased pressure delivery without additional oxygen appears to decrease inflammation (as measured by an inhibition of interferon-gamma release), and delivery of oxygen by mask without any increase in pressure may actually increase inflammation (as measured by an increase in interferon-gamma release) [44]. Since HBOT consists of 2 independent variables (pressure and inspired oxygen concentration), comparison studies are needed in individuals with autism before determining that the clinical effects of 1.3 atm and 24% oxygen are similar to those obtained by delivering oxygen by mask alone without additional pressure. In addition, further studies are needed that evaluate not only the clinical effects of hyperoxia delivered by HBOT, but also the effects of increased atmospheric pressure, because each of these effects may lead to different clinical outcomes depending on the underlying disease pathophysiology.

A primary goal of this study was to determine the effects of HBOT on oxidative stress markers in autistic children. Other objectives were to measure the effects of HBOT on CRP and changes in clinical symptoms. The final intention was to examine the safety of HBOT for use in autistic children. Of note, shorter duration hyperbaric treatment times (45 minutes) were used than what is traditional (60 minutes). This was due, in part, to scheduling constraints.

### Evaluation of the effects of HBOT on oxidative stress markers

Recently, James et al. demonstrated that autistic children had lower levels of plasma reduced (active) GSH and increased levels of oxidized (inactive) GSH when compared to control children [51]. The mean tGSH/GSSG ratio in 73 control children was 28.2 ± 7.0 and in 80 autistic children was 14.7 ± 6.2 (p < 0.0001). The mean fGSH/GSSG ratio was 7.9 ± 3.5 in control children and 4.9 ± 2.2 in the autistic children (p < 0.0001). The mean GSSG in control children was 0.24 ± 0.1 μmol/L and 0.40 ± 0.2 μmol/L in the autistic children (p < 0.0001) [51]. In a previous study, these same researchers demonstrated that the addition of 800 μg folinic acid, 1000 mg of betaine, and 75 μg/kg of injectable methylcobalamin raised tGSH/GSSG in 8 autistic children from 7.5 ± 2.3 to 28.7 ± 7.1 (p = 0.002) and lowered GSSG from 0.59 ± 0.2 nmol/L to 0.25 ± 0.05 nmol/L (p = 0.008). These 8 children had some improvements in speech and cognition, and after these treatments, the levels of tGSH/GSSG and GSSG were both near the levels found in the control children [50].

In the current study, the mean initial tGSH/GSSG was 28.47 ± 4.59 in the 1.3 atm group and 44.68 ± 14.19 in the 1.5 atm group (see Figure 1b). These values are close to or higher than the values found in the control children as described above and are higher than the values described in some autistic children [50,51]. These increased values might be due to the therapies implemented to raise glutathione levels, including folinic acid and methylcobalamin, which many of the children were taking prior to beginning the study. Examination of the 1.3 atm group demonstrates that 7 out of 12 children were taking folinic acid, methylcobalamin, or both. In the 1.5 atm group, 5 out of the 6 children were taking folinic acid, methylcobalamin, or both. Interestingly, analysis of changes in CRP and oxidative stress markers in the children taking these 2 supplements when compared to the children not taking these 2 supplements demonstrated no statistically significant difference in changes in CRP, GSSG, tGSH/GSSG, and fGSH/GSSG (data not shown) at both 1.3 atm and 1.5 atm. In addition, analysis of score changes on the ABC-C, SRS, and ATEC showed no statistically significant difference in the children taking either or both of these 2 supplements when compared to children not taking these (data not shown). In other words, children already taking folinic acid, methylcobalamin, or both had similar changes in markers of oxidative stress, CRP, and clinical outcomes as children not taking these supplements.

In both the 1.3 atm and 1.5 atm groups, after hyperbaric treatment, the ratios of tGSH/GSSG and fGSH/GSSG were both close to the values described by James et al. in control children (see Figure 1b and 1c) and were still higher than the ratios found in most autistic children [51]. Most importantly, from an oxidative stress standpoint, the GSSG levels in both the 1.3 atm and 1.5 atm groups did not significantly change with treatment and were very near to the GSSG levels found in control children (see Figure 1a). Plasma GSSG is a reliable marker of intracellular oxidative stress because it is only exported from cells when intracellular levels exceed the redox capacity. Furthermore, plasma GSSG levels are a better indicator of intracellular oxidative stress than tGSH and fGSH [82]. Therefore, HBOT at the pressures utilized in this study did not appreciably worsen intracellular oxidative stress as measured by changes in plasma GSSG. In addition, there was a trend to lower adenosine levels in the 1.5 atm group (p = 0.078). Elevated adenosine has been described in a subgroup of children with autism and typically leads to elevated S-adenosylhomocysteine (SAH). This is concerning because SAH inhibits most cellular methyltransferases [51]. Therefore, lowering adenosine levels could be of clinical significance in a subgroup of autistic children with elevated adenosine levels.

Even though children in this study had similar changes in oxidative stress markers, CRP, and clinical outcomes whether or not they were taking folinic acid and/or methylcobalamin, therapies to raise glutathione levels in autistic children [50] before initiating HBOT at the pressures used in this study appear prudent. Furthermore, the use of antioxidants [83] might be beneficial in patients with conditions of increased oxidative stress before HBOT is contemplated, especially since antioxidant supplementation is generally recognized as safe. Several antioxidant supplements are known to attenuate oxidative stress induced by higher pressure HBOT (above 2.5 atm) including α-lipoic acid [48], melatonin [84], N-acetylcysteine [85,86], Vitamin E [87], riboflavin [88], selenium [87,88], and glutathione [89]. Furthermore, in two double-blind studies, treatment with an antioxidant, when compared to a placebo, improved behavior in some autistic children [90,91].

### Evaluation of the effects of HBOT on C-reactive protein

Since some autistic children have evidence of neuroinflammation [36-38] and gastrointestinal inflammation [39,40], and since HBOT is known to possess anti-inflammatory properties [43,92] and can decrease both neuroinflammation [42] and gastrointestinal inflammation [46,47], changes in a marker of inflammation were quantified during this study. CRP was chosen (see Figure 2) because it is typically elevated with inflammation [93] and is readily available. In 3 children from the 1.3 atm group with a very high initial CRP, large improvements in mean CRP were found after treatment (p = 0.052). The remaining 9 children in the 1.3 atm group had a small but non-significant improvement of 0.09 mg/L. However, the initial mean CRP in these 9 children was 0.88 mg/L which left little room for improvement. The 1.5 atm group showed an improvement in mean CRP of 0.43 mg/L (p = 0.084). However, since the children in the 1.5 atm group started with low initial CRP levels, dramatic improvements in CRP in these children were not possible. Only those children with an initial high CRP could experience dramatic improvements, which is what was found in this study. Pooling the data for changes in CRP values from all 18 children in this study demonstrated a significant improvement after hyperbaric therapy (p = 0.021). Further evaluation of the effects of hyperbaric therapy on inflammation and inflammatory markers in autistic children, especially at varying pressures and oxygen concentrations, is warranted.

### Evaluation of the effects of HBOT on clinical outcomes

Another outcome of this study was to prospectively examine if the use of hyperbaric therapy led to improvements in clinical symptoms. From our clinical experience with using HBOT in autistic children, some parents have noted improvements in their children. In this study, an inventory of clinical symptoms affected by HBOT was created to help determine if a larger controlled trial was justified, and to investigate which assessment tools might best be utilized in designing a larger study.

The measurements of these clinical outcomes did have some inherent limitations and weaknesses. The use of parent-rated scales and the fact that parents were not blinded to the type of therapy given to their child might have introduced some bias. Furthermore, there was no placebo or control group. Therefore, the improvements found in this open-label study could be due merely to chance or to the natural development of the children. In addition, it is possible that any clinical improvements observed could have occurred as a result of the increased close interaction between the child and parent/caregiver, or motivation and/or enthusiasm that may have developed in the parent/caregiver during the course of the treatments. Because this was a pilot study, the sample sizes were small which makes it difficult to make adequate and meaningful comparisons between the 2 different pressures and oxygen concentrations used. Due to these issues, a larger double-blind, prospective study that includes a control group and more objective outcome measures is warranted.

However, given these limitations, significant improvements in certain areas were found in both the 1.3 atm and the 1.5 atm groups. These improvements were seen in diverse areas including irritability, social withdrawal, hyperactivity, motivation, speech, and sensory/cognitive awareness (see Figures 3 and 4). This range of improvements was somewhat unexpected, but might be explained by the fact that many children with autism have cerebral hypoperfusion which can often vary in location from child to child [35] and correlates anatomically [11] with many core autistic symptoms including repetitive, self-stimulatory behavior [94], and impairments in language [95] and social interaction [34]. It is possible that HBOT might help overcome the effects of cerebral hypoperfusion by providing more oxygen to the brain [21,41], and by causing angiogenesis over time [24,92]. As previously noted, Heuser et al. showed an improvement in cerebral hypoperfusion as measured by SPECT scans in an autistic child after hyperbaric therapy at 1.3 atm [31]. Because HBOT may improve assorted areas of cerebral hypoperfusion, and since these areas may additionally differ in location from child to child, various clinical outcomes could occur. Further research into this area, utilizing HBOT combined with pre- and post-hyperbaric SPECT scans, might be useful in exploring this hypothesis further. A weak trend towards increased inappropriate speech in the 1.3 atm group (see Figure 3a) was observed; this finding was not seen in the 1.5 atm group (see Figure 4a). Further study on the effects of HBOT at 1.3 atm on inappropriate speech is warranted.

### Evaluation of the Safety of HBOT in Children with Autism

The use of HBOT for children is generally regarded as safe, even at pressures of 2.0 atm for 2 hours per day [32]. However, to our knowledge, the safety of HBOT for autistic children had not been previously evaluated. Therefore, throughout each hyperbaric session, the children were intensively monitored. In addition, a parent or caregiver accompanied each child into the chamber, which provided additional monitoring. During this study, no significant adverse events were seen and the treatments were well tolerated. These results suggest that the hyperbaric pressures and oxygen concentrations used in this study are safe in children with autism.

## Conclusion

This prospective open-label pilot study in children with autism indicates, as measured by changes in plasma GSSG, that HBOT ranging from 1.3 to 1.5 atm and 24% to 100% oxygen was not significantly associated with increased intracellular oxidative stress. The use of therapies to raise glutathione levels and lower oxidative stress before beginning HBOT in individuals with autism appears prudent. Among children with high initial CRP, hyperbaric therapy led to a large improvement in CRP levels; this suggests that inflammation in these children improved with treatment. Improvements in clinical outcomes as measured by several scales were observed at both 1.3 atm and 1.5 atm. However, because this study was open-label, conclusions about the efficacy of HBOT as a treatment for autistic children cannot be drawn at this time. Definitive statements regarding the efficacy of HBOT for the treatment of children with autism must await results from future double-blind, controlled trials. Finally, HBOT was safely administered to autistic children in this study, and all participants were able to finish 40 HBOT sessions without any major adverse events.

## List of abbreviations used

ABC-C – Aberrant Behavior Checklist-Community

ATEC – Autism Treatment Evaluation Checklist

atm – Atmosphere

CARS – Childhood Autism Rating Scale

CP – Cerebral palsy

CRP – C-reactive protein

fGSH – Free glutathione

GSH – Glutathione

GSSG – Oxidized glutathione

HBOT – Hyperbaric oxygen therapy

NS – not statistically significant

PDD-NOS – Pervasive Developmental Disorder – Not Otherwise Specified

SAH – S-adenosylhomocysteine

SOD – Superoxide dismutase

SPECT – Single photon emission computed tomography

SRS – Social Responsiveness Scale

tGSH – Total glutathione

## Competing interests

DR, LR, and EM received funding and reimbursement from the International Hyperbarics Association in conjunction with this study. Both DR and EM treat individuals with HBOT in their clinical practices and derive revenue from HBOT. The remaining authors (SJJ and SM) declare that they have no competing interests.

## Authors' contributions

DR and LR conceived of the study and the study design. SJJ and SM carried out the oxidative stress marker analysis. DR, LR, EM oversaw the hyperbaric treatments. DR, SJJ, LR, and EM contributed to the drafting of the manuscript. All authors read and approved the final manuscript.

## Pre-publication history

The pre-publication history for this paper can be accessed here:


